# The effects of orthodontic treatment on personal dental expenditures in South Korea: a follow-up study using Korean health panel survey

**DOI:** 10.1186/s12913-022-09009-8

**Published:** 2022-12-30

**Authors:** Bo-Ra Kim, Han-A Cho, Hosung Shin

**Affiliations:** 1 Department of Dental Hygiene, Namseoul University, Cheonan-si, South Korea; 2 Department of Dental Hygiene, Shinhan University, Uijeongbu-si, South Korea; 3 Department of Social and Humanity in Dentistry, Wonkwang University School of Dentistry, 460 Iksan-daero, Iksan-si, 54538 South Korea

**Keywords:** Oral health, Orthodontics, Out-of-pocket expenditures, Propensity score

## Abstract

**Background:**

This study aimed to investigate the effects of orthodontic treatment on cumulative out-of-pocket (OOP) expenditures for up to 8 years and the factors contributing to changes in individual OOP dental expenses.

**Methods:**

The data of adults aged ≥19 years, 218 with experience of orthodontic treatment (OT group) and 654 without experience of orthodontic treatment (non-OT group) were extracted from the Korea Health Panel Survey between 2009 and 2017 using the propensity score matching method. The total personal OOP expenditure for dental care incurred after orthodontic treatment in the OT group and that incurred in the matched non-OT group were calculated. Since dependent variables, cumulative dental expenditures, were continuous with excess zeros, Tweedie compound Poisson linear models were used to explore the influence of orthodontic treatment experience and demographic and socioeconomic factors, including private insurance, on per capita OOP dental expenditures.

**Results:**

The OT group had socioeconomic characteristics distinct from those of general dental patients. The Box–Cox transformed per capita OOP expenditures for dental care in the OT group were lower than those in the non-OT group (*P* < 0.05). When all covariates were held constant, the non-OT group spent 1.4-times more on OOP dental expenditures, but this was not statistically significant (*P* > 0.1). The data from those with higher incomes revealed the opposite trend (*P* < 0.05), while the other covariates were not statistically significant.

**Conclusions:**

Orthodontic treatment had no positive or negative effect on future oral care use. This finding is similar to the inconsistent results of previous clinical studies on oral health and orthodontic treatment.

## Background

Orthodontic treatment is performed to resolve malocclusions that manifest as symptoms such as mandibular prognathism, facial asymmetry, crowding, or lip fullness [1, 2]. Various treatment methods, including orthodontic surgery, growth observation, and use of fixed orthodontic appliances have been performed in Korea [2, 3]. Regarding the distribution of orthodontic treatment patients who visited a university dental hospital in Korea from 20008 to 2015, approximately 47.5% of the patients were aged between 19 and 39 years, about 47.2% were under 19 years, and approximately 4.4% were ≥40 years [3]. Among them, the psychosocial impact of dental esthetics was found to be more negative for adults seeking orthodontic treatment [4].

Aesthetic improvement after orthodontic treatment is considered to have a positive effect on the recipient’s subjective quality of life [5]. However, analysis of the clinical oral health condition of patients after orthodontic treatment revealed inconclusive results. Root resorption is often the result of a biological response to tooth movement due to increased force [6]. Several studies have also highlighted deterioration of periodontal tissue in patients after orthodontic treatment [7]. In contrast, orthodontic patients have shown improved oral hygiene compared to those with no treatment experience [8], as well as improved health of the gingiva, even during treatment [9]. This may be because correcting malocclusion can improve patients’ oral condition, making such individuals view oral hygiene management more favorably. Another study reported that the caries experience of individuals who had undergone orthodontic treatment did not differ from those who had not received such treatment [10].

The heterogeneity among the findings of previous studies makes it difficult to interpret the effects of orthodontic treatment on oral health. However, the difference in results in some cases may be due to differences in study design rather than the effect of orthodontic treatment itself. For example, varying measurement time points for determining the effectiveness of orthodontic treatment may affect results [11]. Divergences in the health levels of participants and target teeth examined, as well as the absence of a control group [6], make their results inconsistent. Moreover, only a few studies reported the clinical outcomes of orthodontic treatment with sufficient follow-up periods [7, 11].

Oral diseases, which are chronically progressive, require different levels of treatment, depending on their objective and subjective dental needs. However, apart from the need for oral service, access to dental care is often limited by the cost of treatment and is thus affected by the socioeconomic status of the patient. Lower-income and lower education have been associated with fewer dental visits [12, 13] while having dental insurance was reported to be associated with more regular dental visits [14].

Orthodontic treatments are not covered by the National Health Insurance (NHI) in South Korea. NHI has provided benefits of orthodontic treatment only for congenital maxillofacial deformities (cleft lip and nose deformities) to improve masticatory and pronunciation functions since late 2018 [15, 16]. Orthodontic services, with one of their principal purposes being cosmetic improvement [1], have a high proportion of co-payments and a relatively long treatment period. Therefore, the demographic characteristics of patients with this type of service may have unique characteristics that distinguish them from general dental users. The income and education level of patients are also factors associated with the use of orthodontic treatment [17–19]. Disparities in the utilization and expenditure of orthodontic care for American children are greater than those of general and preventive dental care services [20]. As mentioned above, the occurrence, type, and extent of negative results after orthodontic treatment and the treatment costs vary between individuals. Moreover, the use of dental treatment is affected by the socioeconomic status of the patient [6–10, 12–14, 17]. These characteristics may also affect the use of dental services and expenses incurred after the completion of orthodontic treatment. This study aimed to compare the effects of dental treatment costs on individuals who have undergone orthodontic treatment to those without orthodontic treatment experience and to examine the factors affecting the personal cumulative dental expenditure of the study participants.

## Methods

### Data source and study sample

This study used data from the Korea Health Panel (KHP; v.1.6) from 2009 to 2017. The KHP survey is a comprehensive panel survey that was initiated in 2008 and provides nationally representative estimates of healthcare utilization, expenditures, resources, and related influencing factors, including economic activity, income, health behaviors, health status, and private health insurance among individuals and households [21]. Thus, these data were useful in analyzing the patterns of longitudinal dental care use and out-of-pocket (OOP) expenditures. Based on the 2005 Population and Housing Census data, the survey sample households were chosen by a probability proportionate and stratified cluster sampling method using metropolitan cities/provinces and small cities/rural areas as stratification variables. Accordingly, the estimated sample size was approximately 8,000 households nationwide [22]. In 2017, 6,408 households comprising 17,184 individuals were surveyed and annual follow-up data were collected [23].

The study design was approved by the institutional review board of Wonkwang University, South Korea (approval number: WKIRB-202104-SB-019). Informed consent was not required as the KHP data were anonymized by the Korea Institute for Health and Social Affairs. The data of individuals included in the present study were selected from 16,311 individuals who had used dental care services at least once. Adults aged ≥19 years were divided into two groups according to previous orthodontic treatment experience and inclusion criteria as follows: (1) those who completed orthodontic treatment in the cohort inclusion period from 2009 to 2015 (orthodontic treatment [OT] group) or (2) those who did not receive orthodontic treatment at all (non–orthodontic treatment [non-OT] group) for the same cohort inclusion period. Of the 8,198 adults among 16,311 patients, 218 belonged to the OT group. Subsequently, 654 individuals were extracted as the non-OT group, including their sociodemographic characteristics, with follow-up periods similar to those in the OT group. Data from a total of 872 adults were analyzed.

### Variables for analysis

The dependent variable was the per capita OOP expenditures (in South Korean won [KRW]) on dental care. OOP expenditures are the total sum of OOP expenses incurred during the identified follow-up periods (2010–2017), where OOP expenses are the amount paid per visit based on the patient’s outpatient dental care. In the OT group, per capita OOP expenditures for dental care were calculated by adding the OOP expenditures from the year following orthodontic treatment to 2017. The OOP expenditures of the non-OT group were calculated by totaling the OOP for dental care spending during the same follow-up period as that used in the OT group.

**Fig. 1:**
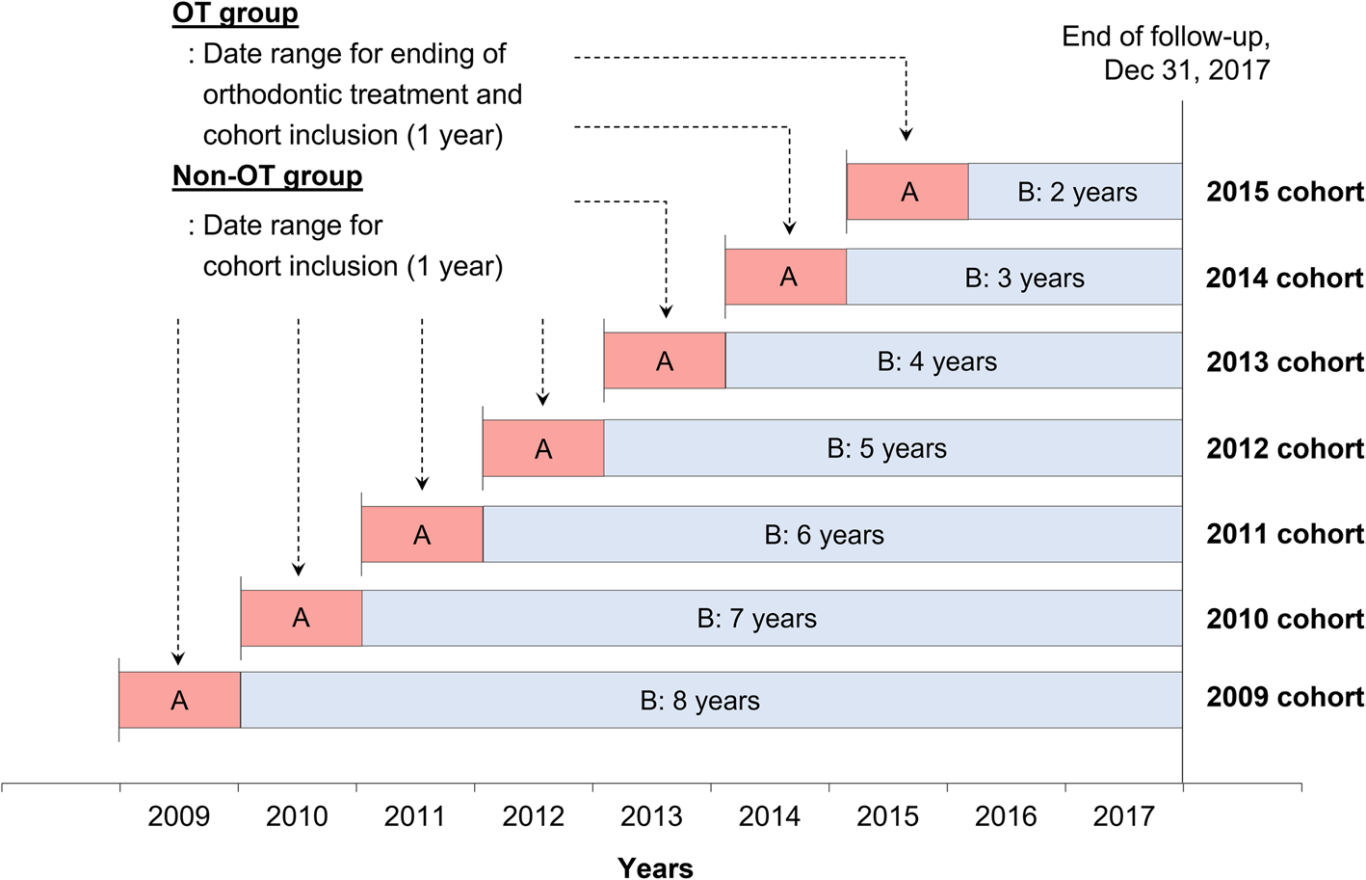
Study schematic showing follow-up periods for the OT and non-OT groups in each yearly cohort. **A** is the date range of completion of orthodontic treatment and the cohort inclusion period of the OT and non-OT groups. **B** represents the follow-up periods for calculating per-capita OOP expenditures for dental care of both groups in each yearly cohort

The follow-up period was different for each cohort. For example, in the 2009 cohort, the OT group that completed orthodontic treatment in 2009 had a follow-up period of eight years from 2010 to 2017. The corresponding control group, that is, the non-OT group of the 2009 cohort, included the same follow-up periods as the OT group (Fig. 1). In the 2015 cohort, the OT and non-OT groups had two years of follow-up, from 2016 to 2017.

To analyze the impact of orthodontic treatment experiences and demographic and socioeconomic characteristics on the per capita OOP expenditures for dental care, the following covariates were used: sex (male and female), age (19–29 and ≥30 years), employment (yes/no or do not know), education level (≤ high school and ≥ college), marital status (yes/no), private insurance status (yes/no), equivalent income level (low, middle, and high), and duration of the follow-up period (2–8 years). Age was classified based on the age at which people get married because the group with the greatest demand for orthodontic services in South Korea comprises single individuals (the average age for Korean women to get married is between 30 and 31 years [24]). Equivalent income (unit: 10 million KRW) was defined as an equivalized household income obtained by dividing the total household income by the square root of household size for one household member [25]. Equivalent income was divided into three groups by quantile (low: 1st quintile, Q1; middle: 2nd–4th quintiles, Q2–Q4; high: 5th quintile, Q5).

### Statistical analysis

Propensity scores (PS) were used to match individuals in the non-OT group with demographic and socioeconomic characteristics similar to those in the OT group. Triploid individuals (*n* = 654) were extracted for the non-OT group to improve statistical power. The covariates for PS were as follows: sex; age (10 categories based on units of 5 years); education (≤ elementary school, middle and high school, or ≥college); type of insurance (work, region, medical aid, or other); employment, equivalent income quintiles (Q1 = lowest and Q5 = highest; unit: KRW); marital status; private insurance status; and follow-up periods (2–8 years). The nearest-neighbor method was used for the matching algorithm. Balanced diagnostics before and after PS matching were verified to ensure that the standard mean difference (SMD) between the two groups for each covariate was <0.1.

The distribution of individuals between the OT and non-OT groups according to demographic and socioeconomic factors was compared using the chi-squared test. Since the personal OOP expenditures for dental care were zero-inflated and skewed to the right, Box–Cox transformation and t-test were used to compare the personal OOP dental expenditures between the two groups. The Tweedie compound Poisson linear model was used to analyze the impact of orthodontic treatment experience and demographic and socioeconomic variables on personal OOP dental care expenditures [26]. The authors did not consider analytical models suited for results from two separate data-generation processes (e.g., two-part model or Tobit model [27]) because the data-generation process of both true zeros and non-zero groups was performed in a single process in this study. All statistical analyses were performed using R software (v.4.1.2; R Foundation for Statistical Computing, Vienna, Austria).

## Results

The distribution of the study participants before and after PS matching is presented in Table 1. Before matching, 7,902 adults met the inclusion criteria and had undergone no orthodontic treatment according to the KHP data, showing a different distribution compared to the 218 adults in the OT group for all covariates. After matching, 654 adults were selected as the non-OT group and the distributions between the two PS-matched groups for all covariates were similar.

**Table 1:** Distribution of participants before and after propensity-score matching

Variables	OT group	Non–OT group	
	Before and after matching (*n* = 218)	Before matching (*n* = 7902)	After matching (*n* = 654)
	*n* (%)	*n* (%)	*n* (%)
Sex			
Male	50 (22.9)	3569 (45.2)	165 (25.2)
Female	168 (77.1)	4333 (54.8)	489 (74.8)
Age (years)			
19–24	109 (50.0)	530 ( 6.7)	313 (47.9)
25–29	51 (23.4)	385 ( 4.9)	174 (26.6)
30–34	27 (12.4)	432 ( 5.5)	80 (12.2)
35–39	9 ( 4.1)	643 ( 8.1)	24 ( 3.7)
40–44	10 ( 4.6)	815 (10.3)	29 ( 4.4)
45–49	7 ( 3.2)	825 (10.4)	22 ( 3.4)
50–54	4 ( 1.8)	791 (10.0)	9 ( 1.4)
55–59	0 ( 0.0)	797 (10.1)	0 ( 0.0)
60–64	0 ( 0.0)	691 ( 8.7)	0 ( 0.0)
≥65	1 ( 0.5)	1993 (25.2)	3 ( 0.5)
Follow-up period (years)			
2	37 (17.0)	873 (11.0)	93 (14.2)
3	38 (17.4)	1420 (18.0)	137 (20.9)
4	34 (15.6)	744 ( 9.4)	97 (14.8)
5	30 (13.8)	758 ( 9.6)	84 (12.8)
6	29 (13.3)	1025 (13.0)	81 (12.4)
7	32 (14.7)	1320 (16.7)	107 (16.4)
8	18 ( 8.3)	1762 (22.3)	55 ( 8.4)
Education level			
≤ Elementary school	1 ( 0.5)	1662 (21.0)	3 ( 0.5)
Middle and high school	41 (18.8)	3476 (44.0)	128 (19.6)
≥ College	176 (80.7)	2764 (35.0)	523 (80.0)
Health insurance			
Work	158 (72.5)	5382 (68.1)	474 (72.5)
Region	58 (26.6)	2153 (27.2)	173 (26.5)
Medical aid	1 ( 0.5)	292 ( 3.7)	5 ( 0.8)
Others	1 ( 0.5)	75 ( 0.9)	2 ( 0.3)
Employment			
No/do not know	127 (58.3)	3256 (41.2)	378 (57.8)
Yes	91 (41.7)	4646 (58.8)	276 (42.2)
Equivalent income level			
Q1 (lowest)	11 ( 5.0)	1613 (20.4)	38 ( 5.8)
Q2	34 (15.6)	1590 (20.1)	97 (14.8)
Q3	36 (16.5)	1588 (20.1)	115 (17.6)
Q4	69 (31.7)	1555 (19.7)	205 (31.3)
Q5 (highest)	68 (31.2)	1556 (19.7)	199 (30.4)
Marital status			
No	171 (78.4)	2154 (27.3)	512 (78.3)
Yes	47 (21.6)	5748 (72.7)	142 (21.7)
Private insurance			
No	50 (22.9)	2380 (30.1)	146 (22.3)
Yes	168 (77.1)	5522 (69.9)	508 (77.7)

*OT* Orthodontic treatment, *Non-OT* Non-orthodontic treatment

*Q1* First quintile (lowest), *Q2* Second quintile, *Q3* Third quintile, *Q4* Fourth quintile, *Q5* Fifth quintile (highest)

**Fig. 2:**
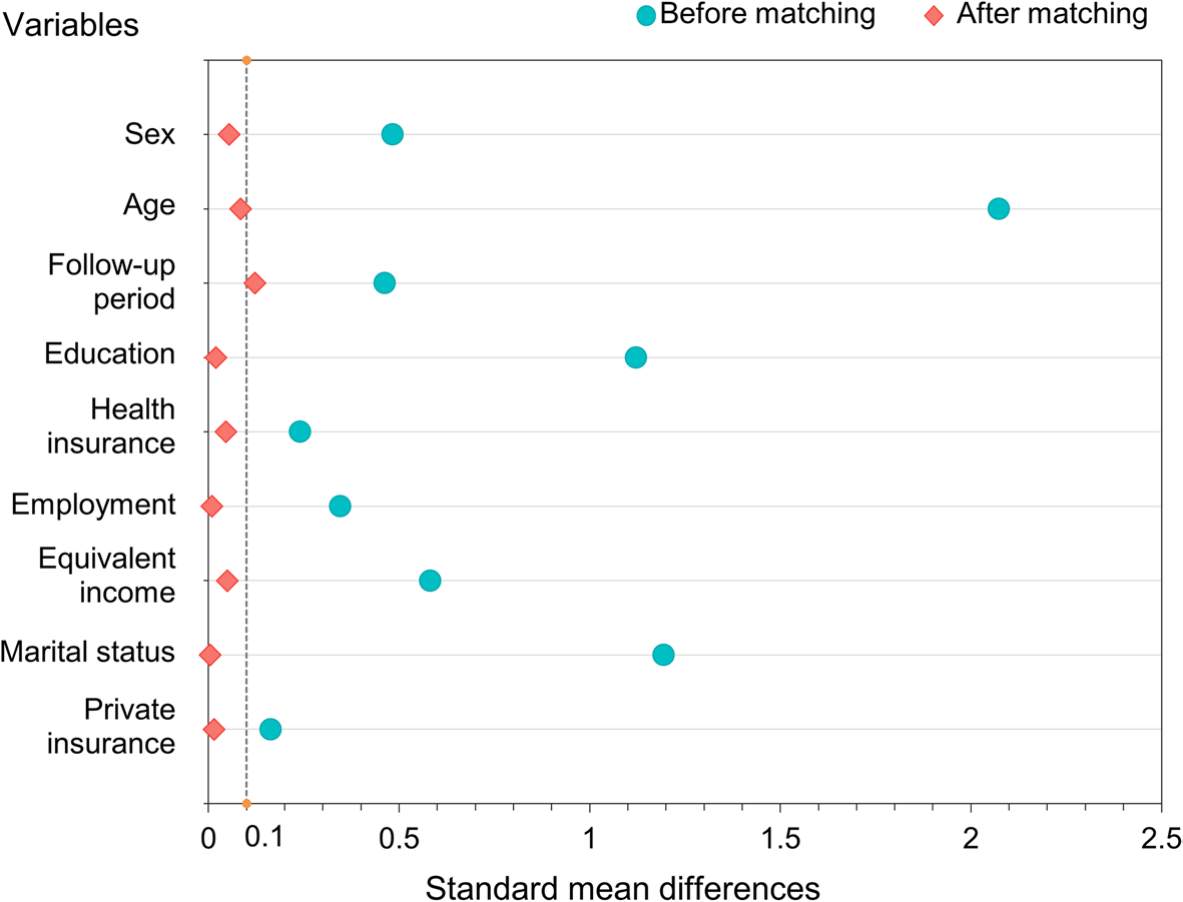
Plot of standard mean difference for each covariate before and after propensity score matching

The distribution of SMD for each covariate obtained before and after PS matching is shown in Fig. 2. After matching, the SMDs of all covariates except for the variable for the follow-up periods (SMD = 0.122) were <0.1, representing the balance of the covariates between the two groups.

Table 2 presents the distribution of all variables recategorized for the final analysis model. Participants who showed the unique characteristics of orthodontic treatment users showed a different distribution compared to general dental care users. More than 70% of the study participants were women, 19–29 years old, had a college degree or higher, were single, or had private insurance. The smallest portion was the low-income group, with approximately 5%, and the largest portion was the middle-income group (64%). The mean age of the study participants (*N* = 872) was 27.20 years (standard deviation = 8.03).

**Table 2:** The Box–Cox transformed per-capita OOP dental expenditures and distribution of both groups by covariates

Variables	OT group (*n* = 218)	Non-OT group (*n* = 654)	*P*-value^*^
	*n* (%)	*n* (%)	
Per capita OOP dental expenditures(Unit: KRW)	9488 (22868)	13448 (31154)	0.045^a^
Sex			
Male	50 (22.94)	165 (25.23)	0.496
Female	168 (77.06)	489 (74.77)	
Age (years)			
19–29	160 (73.39)	487 (74.46)	0. 496
≥ 30	58 (26.61)	167 (25.54)	
Education level			
≤ High school	42 (19.27)	131 (20.03)	0.806
≥ College	176 (80.73)	523 (79.97)	
Employment			
No/do not know	127 (58.26)	378 (57.80)	0.905
Yes	91 (41.74)	276 (42.20)	
Income level			
Low (Q1)	11 ( 5.05)	38 ( 5.81)	0.904
Middle (Q2–Q4)	139 (63.76)	417 (63.76)	
High (Q5)	68 (31.19)	199 (30.43)	
Marital status			
No	171 (78.44)	512 (78.29)	0.962
Yes	47 (21.56)	142 (21.71)	
Private insurance			
No	50 (22.94)	146 (22.32)	0.851
Yes	168 (77.06)	508 (77.68)	

*OT* Orthodontic treatment, *Non-OT* Non-orthodontic treatment

*Q1* 1st quintile, *Q2–Q4* 2nd–4th quintile, *Q5* 5th quintile

^a^Per capita OOP expenditure denotes mean (standard deviation); *p*-value was obtained using a t-test

^*^P-values for covariates were obtained using a chi-squared test excepting for mean per capita OOP dental expenditures

**Fig. 3:**
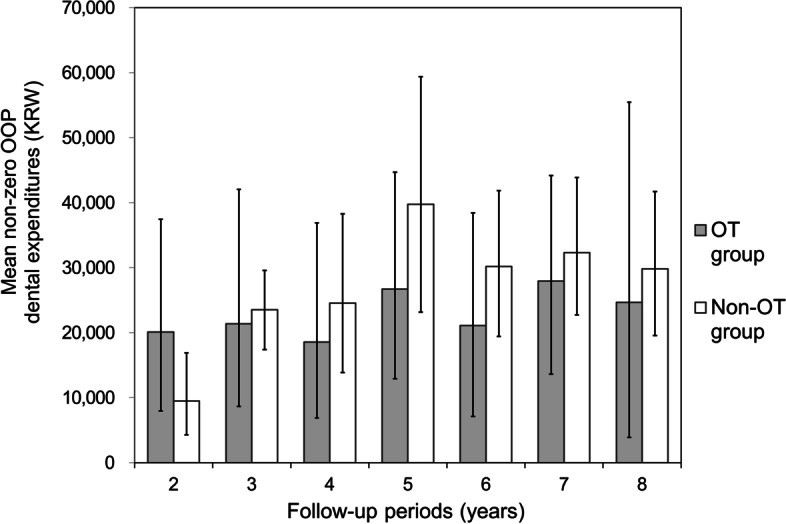
Cox–Box transformed per-capita non-zero OOP dental expenditures by follow-up period. Mean values and 95% confidential intervals showed no significant differences between the OT and non-OT groups in all yearly cohorts (unit: KRW)

Regardless of the follow-up period, the mean value of the Box–Cox transformed per capita OOP expenditures for dental care of the OT group was smaller than that of the non-OT group, which was a statistically significant (*P* = 0.045, Table 2). A high proportion of participants with zero OOP expenditures for dental care was observed in the two groups—59% in the OT group and 52% in the non-OT group. Fig. 3 shows the Cox-Box transformed per capita non-zero OOP expenditures for dental care according to the follow-up periods. In general, the cumulative expenditures of the OT group were lower than those of the non-OT group, but no significant difference was observed between the two groups (*P* > 0.05). A significant linear time trend was observed in both total and non-zero expenditures (*P* < 0.05), but no statistically significant linear time trend was observed in either the OT group or the non-OT group individually (*P* > 0.25).

The results of the Tweedie compound Poisson linear model are summarized in Table 3. When all other covariates were adjusted, the non-OT group showed 1.397-times higher OOP expenditures per capita for dental care than the OT group, but no statistical significance was observed. Individuals in the middle-income group were likely to pay less in OOP dental expenditures than those in the low-income group (exp.[coef.] = 0.429, *P* = 0.026). High-income individuals showed a marginally significant result (exp.[coef.] = 0.457, *P* = 0.054). Overall, per capita OOP dental expenditures were likely to increase with longer follow-up periods.

**Table 3:** Tweedie compound Poisson linear model for the effect of variables on per-capita OOP dental expenditures

	Coefficient	Exp. (coef.)	*P*-value
Orthodontic treatment			
Yes	Ref.		
No	0.335	1.397	0.134
Sex			
Male	Ref.		
Female	0.327	1.386	0.149
Age (years)			
19–29	Ref.		
≥30	0.010	1.010	0.977
Education			
≤High school	Ref.		
≥College	0.134	1.144	0.581
Employment			
No/do not know	Ref.		
Yes	0.128	1.137	0.542
Income level			
Low (Q1)	Ref.		
Middle (Q2–Q4)	-0.846	0.429	0.026
High (Q5)	-0.782	0.457	0.054
Marital status			
No	Ref.		
Yes	-0.082	0.921	0.813
Private insurance			
No	Ref.		
Yes	-0.021	0.979	0.927
Follow-up period			
(years)	0.180	1.197	<0.001

*Q1* 1st quintile, *Q2–Q4* 2nd–4th quintile, *Q5* 5th quintile

*Exp.* Exponential, *Coef.* Coefficient, *95 % CI* Confidence interval (95%)

## Discussion

The current study attempted to analyze how the orthodontic treatment experiences of adults affected their per capita OOP expenditures for dental care using the KHP survey data over a two-to eight-year period. Resultantly, the orthodontic treatment experience showed no statistical significance regarding individual OOP dental expenditures. This was contrary to the results of previous studies that reported a positive relationship due to the changes in oral conditions during and after orthodontic treatment.

The rearrangement of dentition by orthodontic treatment can result in a lower prevalence of periodontitis or caries experience by helping resolve the causes of oral health [28–30]. Progress in orthodontic treatment requires regular dental visits, thus providing opportunities to detect oral diseases early or deal with other dental problems. Moreover, professional plaque control might be performed more often during orthodontic treatment to compensate for the difficulty in meticulous oral hygiene practice by patients due to intraoral orthodontic appliances [17]. Additionally, orthodontic patients receive continuous oral health and oral hygiene education through regular visits during orthodontic treatment. This allows the patients to recognize the risk of poor oral hygiene and oral diseases and to change their dental behaviors [31, 32]. Thus, the experience of orthodontic care motivates individuals to improve their oral health [33, 34]. This could elicit fewer new oral diseases and help individuals maintain better oral health after completing orthodontic treatment over time. However, the attitude that occurred during orthodontic treatment may disappear and may not be maintained after the end of orthodontic treatment. A recent study reported that the experience of fixed orthodontic treatment in childhood and the accompanying regular treatment may not affect adult dental knowledge and behaviors [34].

Orthodontic treatment is performed not only for the therapeutic purpose of malocclusion but also for esthetic improvement [1]. Therefore, the orthodontic treatment group in our study had characteristics different from those of general dental care users, such as sex, income level, and education level [17–19, 35–37]. In the US, dental visits and OOP costs for orthodontic treatment fluctuate more significantly with economic downturns and recoveries [38]. In Korea, the proportion of orthodontic treatment paid OOP to total dental expenses was higher in the highest income bracket than that in the lowest-income decile [23]. In general, orthodontic treatment is expensive and co-payment rates are high. Therefore, those who undergo orthodontic treatment are often people who can afford the OOP expenses. The participants of this study included those who had undergone orthodontic treatment (OT group) and a control group (non-OT group) matched by their characteristics. Of these, more than 60% belonged to the relatively high-income quintiles (quintiles 4–5). Therefore, caution is needed in interpreting the results or in comparison with previous studies, in that the results were analyzed for groups with relatively high-income levels.

In this study, individuals with higher incomes were likely to spend less per capita on OOP expenditures for dental care. This was contrary to the expected results according to previous studies, which showed that households with high income levels tended to spend more OOP on dental care [36, 39]. In the short term, lower income groups may have higher unmet needs and worse oral health, which requires expensive procedures [40, 41]. This is mainly because low-income acts as a barrier to regular dental visits and associated expenses for patients [42, 43]. An Australian study implementing a self-reported format found that poorer oral health was associated with higher total dental expenditures over 12 months [44]. From a long-term perspective, recent evidence in the literature [45] has indicated that greater social position, as determined by property status, education, and income level, is directly related to less tooth loss and less poor self-reported oral health status.

Unlike income level, private insurance was not associated with per capita OOP expenditures for dental care. Private insurance tends to be purchased more by high-income households and is one of the factors driving high dental care use [13, 25, 46, 47]. Public coverage for dental expenditures is largely limited, with only approximately 30% of costs covered by the government or compulsory insurance in developed countries [48]. Consequentially, individuals with private dental insurance may have less burden in bearing OOP expenditures and may be more able to afford regular dental visits [44]. Accordingly, it was predicted that private insurance subscribers would use more dental services [49, 50]. The OOP dental costs that patients actually pay after treatment would not generally be high given that private insurance reimburses dental expenses [44, 46]. Although the private insurance subscription rate of participants in this study was considerably high, at more than three-quarters (77%) of all participants, the difference in dental OOP expenses between those who were and were not privately insured was not statistically significant.

This study had some limitations. First, individuals aged ≤18 years were excluded considering mixed dentition because orthodontic treatment duration could vary depending on the time of loss of primary teeth and eruption of permanent teeth. The number of participants aged 19–29 was approximately three times higher than that aged ≥30 years. This indicates that treatment mainly occurred disproportionately in younger adults. Dental visits for orthodontic treatment in children and adolescents increased from 12% in 2008 to 20% in 2013 in South Korea [51]. Therefore, further studies need to be expanded to include younger age groups.

Second, SMD for the duration of the follow-up period variable was 0.122, slightly larger than that of other socioeconomic variables (threshold = 0.1) in the PS matching analysis, which may have affected the matching results (Fig. 2). The earlier orthodontic treatment starts in one’s life, the greater the total dental expenditure. For this reason, this study used the duration variable in the PS matching analysis.

In addition, this study has some limitations stemming from the use of survey data. First, the OOP expenses in KHP data were coded based on the dental expenses received by the study participants. However, some receipts might have been dropped because of problems with some participants. Second, the dental treatment history or corresponding OOP expenses surveyed by the study participant do not reflect the objective and overall oral health status at that time. To overcome the lack of the information on baseline oral health status, this study designated the end point of orthodontic treatment as the starting point of follow-up. Moreover, the control group (non-OT) was selected by matching individuals with socioeconomic characteristics similar to those of the orthodontic treatment group.

## Conclusions

Individuals’ experience of orthodontic treatment did not affect the increase or decrease in expenditures on dental treatment for the next two-eight years. Therefore, the effects of orthodontic treatment evaluated by dental care expenditures do not appear to contribute to either the improvement or the deterioration of oral health; this was similar to the findings of previous clinical studies. This study also found that in the group with unique demographic and socioeconomic characteristics for orthodontic treatment, higher equivalized household income was associated with lower OOP dental expenditures.

## Data Availability

The data that support the findings of this study are available from the Korea Institute for Health and Social Affairs, but restrictions apply to the availability of these data, which were used under license for the current study and so are not publicly available. However, data are available from the authors upon reasonable request and with permission of the Korea Institute for Health and Social Affairs. The datasets are available at https://www.khp.re.kr:444/eng/main.do.

## Abbreviations

KHP: Korea Health Panel
OOP: Out-of-pocket
OT: Orthodontic
non-OT: Non-orthodontic
KRW: South Korean won
Q1: 1st quintile
Q2: 2nd quintile
Q3: 3rd quintile
Q4: 4th quintile
Q5: 5th quintile
PS: Propensity score
SMD: Standard mean difference
SD: Standard deviation
CI: Confidence interval
Exp.: Exponential
Coef.: Coefficient
